# Multivariable risk prediction can greatly enhance the statistical power of clinical trial subgroup analysis

**DOI:** 10.1186/1471-2288-6-18

**Published:** 2006-04-13

**Authors:** Rodney A Hayward, David M Kent, Sandeep Vijan, Timothy P Hofer

**Affiliations:** 1 Department of Veterans Affairs, VA Center for Practice Management & Outcomes Research, VA Ann Arbor Healthcare System, Ann Arbor, MI, USA; 2 Department of Internal Medicine & Michigan Diabetes Research & Training Center, University of Michigan School of Medicine, Ann Arbor, MI, USA; 3 Division of Clinical Care Research, Department of Medicine, Tufts-New England Medical Center and Tufts University School of Medicine, Boston, MA, USA; 4 The Robert Wood Johnson Clinical Scholars Program, University of Michigan School of Medicine, Ann Arbor, MI, USA

## Abstract

**Background:**

When subgroup analyses of a positive clinical trial are unrevealing, such findings are commonly used to argue that the treatment's benefits apply to the entire study population; however, such analyses are often limited by poor statistical power. Multivariable risk-stratified analysis has been proposed as an important advance in investigating heterogeneity in treatment benefits, yet no one has conducted a systematic statistical examination of circumstances influencing the relative merits of this approach vs. conventional subgroup analysis.

**Methods:**

Using simulated clinical trials in which the probability of outcomes in individual patients was stochastically determined by the presence of risk factors and the effects of treatment, we examined the relative merits of a conventional vs. a "risk-stratified" subgroup analysis under a variety of circumstances in which there is a small amount of uniformly distributed treatment-related harm. The statistical power to detect treatment-effect heterogeneity was calculated for risk-stratified and conventional subgroup analysis while varying: 1) the number, prevalence and odds ratios of individual risk factors for risk in the absence of treatment, 2) the predictiveness of the multivariable risk model (including the accuracy of its weights), 3) the degree of treatment-related harm, and 5) the average untreated risk of the study population.

**Results:**

Conventional subgroup analysis (in which single patient attributes are evaluated "one-at-a-time") had at best moderate statistical power (30% to 45%) to detect variation in a treatment's net relative risk reduction resulting from treatment-related harm, even under optimal circumstances (overall statistical power of the study was good and treatment-effect heterogeneity was evaluated across a major risk factor [OR = 3]). In some instances a multi-variable risk-stratified approach also had low to moderate statistical power (especially when the multivariable risk prediction tool had low discrimination). However, a multivariable risk-stratified approach can have excellent statistical power to detect heterogeneity in net treatment benefit under a wide variety of circumstances, instances under which conventional subgroup analysis has poor statistical power.

**Conclusion:**

These results suggest that under many likely scenarios, a multivariable risk-stratified approach will have substantially greater statistical power than conventional subgroup analysis for detecting heterogeneity in treatment benefits and safety related to previously unidentified treatment-related harm. Subgroup analyses must always be well-justified and interpreted with care, and conventional subgroup analyses can be useful under some circumstances; however, clinical trial reporting should include a multivariable risk-stratified analysis when an adequate externally-developed risk prediction tool is available.

## Background

Although many types of "evidence" can help guide clinical practice, [1-3] the randomized controlled trial (RCT) is the standard by which we define "evidence-based" medicine and is the preferred "evidence" when setting guidelines and policies for patient care [4-8]. It is well known, however, that the average results of RCT's often do not apply to all, or even most, individuals in a clinical trial, since a small group of patients who receive substantial benefit can heavily influence the *average *benefit (i.e., mean main effect) across all study subjects [2,3,9-14]. Therefore, subgroup analysis is often used in an attempt to identify specific groups of patients who may receive substantially more or less benefit compared to the average effect of the study intervention[15].

Differences in the *absolute *benefit of treatment across subjects arises even when a treatment has a homogeneous net *relative *risk reduction (RRR), since in such instances absolute risk reduction (ARR) will vary as a function of a person's risk in the absence of treatment [2,9,10,14,16-20]. For example, if the RRR is 50% in all study subjects, treating someone with a 40% 5-year risk will result in a 20 in 100 ARR (number needed to treat [NNT] = 5) but someone with a 4% risk will receive only a 2 in 100 ARR (NNT = 50)[20]. This heterogeneity in ARR will be further amplified any time that there is treatment-related harm that is, at least in part, independent of risk for the outcome in the absence of treatment. There are many known examples of this phenomenon. For example, amongst middle-aged men, higher and lower cardiovascular (CV) risk patients have similar risks of bleeding complications from aspirin therapy. Therefore, as CV risk in the absence of treatment decreases, at some point harm related to aspirin therapy will exceed that of the benefits of aspirin therapy. This phenomenon is almost ubiquitous when very low-risk individuals can be identified, since most interventions have a non-negligible risk that is largely unrelated to the subject's risk for the targeted adverse outcome.

Conventional subgroup analysis may be poorly suited to detect such heterogeneity in treatment benefit [2,9,11-15,22,23]. In particular, conventional subgroup analysis often has quite limited statistical power [15-21]. Since there are usually multiple variables that merit subgroup comparisons, the risk of false positive findings due to multiple comparisons compounds the risk of false negative findings due to low statistical power.

Multivariable risk-stratified analysis, an approach to subgroup analysis that utilizes multivariable prediction tools, has been advocated by some methodologists as a supplement to or replacement for conventional subgroup analysis [2,9,11,14,22,23]. A multivariable risk-stratified approach does not conduct multivariable analysis directly on the clinical trial data, but rather, risk-stratifies the study population based upon their known risk factors using a multivariable risk prediction tool, which should be developed and validated using previous observational and experimental studies[22]. The multivariable prediction tool is used to calculate each study subject's predicted risks and benefits from treatment; then, a single statistical comparison is conducted to test for heterogeneity in net treatment benefit (estimated treatment-related benefit *minus *estimated treatment-related harm).

Some recent studies have used this type of multivariable risk-stratified analysis and have uncovered major variations in net relative treatment benefit that were not identifiable by examining patient factors one at a time. For example, The Global Utilization of Streptokinase and tPA for Occluded coronary arteries (GUSTO) study, published in 1993, found a statistically and clinically significant decrease in mortality for acute myocardial infarction in patients who were treated with accelerated tPA compared to those treated with streptokinase ($33,000 per life-year saved) and traditional subgroup analyses did not accurately identify subgroups that did not benefit[24]. However, Kent et al[22] reanalyzed the GUSTO results but this time stratified patients based upon an externally developed and validated model (a model not available at the time of the original report) that predicted: 1) risk of death due to myocardial infarction, 2) risk of thrombolytic-related intracranial hemorrhage, and 3) differential benefit from thrombolytics (as determined by time from onset of chest pain to the time of thrombolytic administration). They found that 25% of GUSTO subjects accounted for more than 60% of the net benefit ($13,900 per life-year saved) and that almost 100% of the net benefit of tPA occurred in the half of patients with the highest predicted benefit. No single variable or simple combination of variables was able to discriminate between patients most likely or unlikely to benefit from tPA. Although the number of published risk-stratified analyses of clinical trials remain few in number, similar anecdotes regarding the superiority of a risk-stratified analysis have been reported for other cardiac treatments[25] and for surgical and medical treatments directed at stroke prevention and therapy [26,27]. In most of these examples, conventional subgroup analyses were unable to identify large variations in treatment benefit that were identifiable using multivariable risk-stratification.

All sub-group analyses require cautious interpretation; [15,21] however, there are several proposed reasons why a multivariable risk-stratified approach might be a major improvement on traditional subgroup analysis. First, combining predictors of net benefit (risk factors for bad outcomes in the absence of vs. in the presence of the treatment) into a single prediction tool can greatly increase the degree of risk-stratification, since most common health outcomes have multiple independent risk factors [22,28-40]. Examples include predicting cardiovascular risk using a single risk factor vs. using the Framingham 10-year risk calculator[39] or predicting ICU death using any 1 or 2 of APACHE's components vs. the full APACHE III model[36]. Second, prediction tools often produce continuous estimates of risk. Previous work by Brookes et al [21,41] has demonstrated how subgroup analyses that utilize continuous independent variables and examine interaction terms can produce substantial improvements in statistical power, since such analytic approaches better utilize the full sample size of the study by avoiding dividing the sample up into discrete subgroups[22]. Finally, a multivariable risk-stratified analysis reduces the chance of false positive results because it represents a single statistical comparison (thereby avoiding the multiple comparisons of conventional subgroup analysis).

Although recent commentaries have advocated that multivariable risk-stratification should be employed more routinely, [2,9,11,14,22] we can find no systematic statistical evaluations trying to quantify the relative benefits of a multivariable approach over conventional one-variable-at-a-time subgroup analysis and circumstances that impact their relative statistical power when trying to detect heterogeneity in net relative risk reduction related to treatment-related harm. Therefore in this paper we examine the statistical power obtained using traditional vs. risk-stratified subgroup analyses under a variety of commonly occurring study circumstances.

## Methods

There are 3 factors that can influence whether one subgroup benefits more or less from an intervention than another: 1) greater risk of adverse outcomes in the absence of treatment (predictors of pre-treatment risk), 2) greater probability of treatment complications (predictors of treatment-related risk), and 3) greater chance of benefiting from treatment (predictors of differential relative treatment response) [22]. In this paper, we considered a simplified case in which only predictors of pre-treatment risk are known, which can be estimated in a clinical trial by examining the event rate in the control group). There are three reasons for choosing this approach. First, it is the most common scenario. Unlike genetically engineered mice used in laboratory experiments, people in most clinical trials have substantial heterogeneity in the number and type of risk factors present at baseline. There exist published multivariable risk prediction tools for most common major clinical outcomes, allowing us to risk-stratify people, at least to some degree, into subjects with lower vs. higher risk in the absence of the study treatment [1,28-40] (although the predictiveness of these tools may vary when used in different clinical situations and patient populations). In contrast, risk prediction tools for treatment-related complications are not as common. Further, we know even less about biologic factors that may influence heterogeneity in subject's relative response to treatment (although this may change in the future as genetic mechanisms of treatment response are better delineated). Second, varying parameters across all three domains simultaneously (pre-treatment risk, treatment-related risk and differential relative treatment response) requires the combination of multiple models which greatly increases the complexity of presenting and understanding what is influencing the results. Since the basic principle is the same whether you vary all three factors or just pre-treatment risk, we felt that the increased complexity of varying all three factors was not justified, especially since varying only pre-treatment risk leads to a conservative estimate of the degree of treatment-effect heterogeneity and our results were already quite favorable to a multivariable risk-stratified approach,.

Therefore, we examined the relative merits of conventional sub-group vs. risk-stratified subgroup analysis under a scenario in which there is a small annual probability of treatment-related complications that is independent of pre-treatment risk for the study's primary outcome. Clinical examples include heterogeneity in the treatment-effect of anti-coagulation for atrial fibrillation (since the risk factors for anticoagulant-related bleeding complications differ from the risk factors for stroke), and heterogeneity in the benefits of aortic aneurysm surgery (since the risk factors for the surgery differ from the risk factors for aneurysm rupture).

In all examples reported in this paper, the study has the following basic characteristics:

1. N = 8800 (n_1 _= 4400 and n_2 _= 4400), yielding 80% statistical power to detect a relative risk reduction of ≥ 25% (two-tailed test; α = 0.05), and

2. The treatment decreases pre-treatment risk of major adverse events (i.e., the control event rate [CER]) by 50% over a 5-year period but at a cost of some uniform rate of treatment related harm.

**Figure 1:**
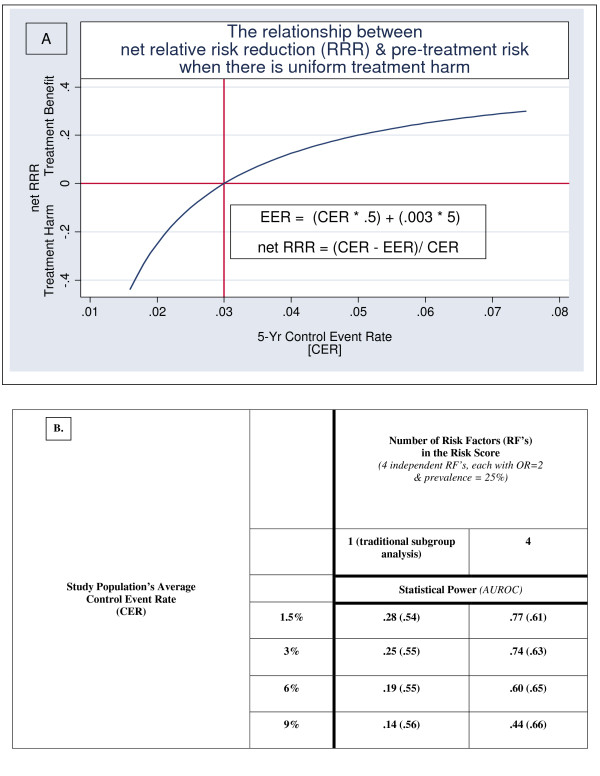
Panel A shows how overall treatment benefit (net RRR is a function of [treatment benefit] – [treatment harm]) varies as a function of pretreatment risk (as estimated by the control event rate [CER] of an RCT) when a treatment decreases pre-treatment risk by 50% but at a cost of 0.003 treatment-related adverse events per treatment-year. As a result, lower risk patients are harmed by treatment and higher risk patients benefit from treatment. Panel B demonstrates two statistical phenomena: (1) that statistical power can be greatly enhanced by combining risk factors (RF's) into a risk index, and (2) statistical power is greatest when the study population includes more low risk patients

The base-case sets the treatment-related harm at 3 serious treatment complications per year per 1000 patients treated (0.3%). This results in a net treatment benefit determined by the formula: net 5-year RRR = [control event rate * .5] - [0.003 * 5]). This basic scenario provides us with an example of a well-powered study for detecting main effects in which the medication is very effective in decreasing the risk of some bad outcomes but has substantial variation in the treatment's net RRR (treatment-related benefit *minus *treatment-related harm) due to a small uniform rate of treatment-related harm (see Figure 1).

Next, we examined the statistical power of conventional and multivariable risk-stratified analysis to detect the presence of this treatment-effect heterogeneity under a variety of study circumstances. First, we examined the impact of varying the average pre-treatment risk of the study population (CERs) and the number of independent risk factors. We then examined the impact of the following on conventional subgroup analysis: changes in 1) the risk factor's odds ratio, and 2) the prevalence of the individual risk factor being examined. Finally, we examined the impact of the following on a multivariable risk-stratified approach: 1) the number, prevalence and odds ratios of the individual risk factors in the prediction model, 2) the predictiveness of the model, 3) the accuracy of the prediction model's weights, and 4) the probability of treatment-related complications. The predictiveness of the multivariable tool was summarized by the area under the receiver operator characteristic (AUROC) curve from a logistic regression model, also known as the C-statistic.

For each of the instances above, we calculated the statistical power based upon standard simulation techniques[41]. When trying to represent the complexities of the real world, there is often not an appropriate analytic method or closed form expression for determining sample size requirements. Under such circumstances, simulation is the best method for exploring important questions about study design and power [21,41-44]. Simulation techniques can allow for consideration of the heterogeneity of treatment effects and interactions and correlations between patient factors. For each iteration of the simulation, a sample was generated by randomly drawing observations, with replacement, from a population with the specified study characteristics. The parameters were estimated by logistic regression with the occurrence of the primary outcome (1 = yes, 0 = no) as the dependent variable for each of two thousand iterations of the specified sample size (N = 8800). The overall treatment effect was tested using treatment arm (1 = treated, 0 = control) as the independent variable. The significance of subgroup effects were tested using an interaction term, as proposed by Brookes, et al [21,41]. This model included a variable for the subgroup (either for the individual risk factor [RF] or a multivariable risk index), the variable for treatment arm and an interaction term between the two (RF*intervention_group) in the regression model. A test statistic was calculated for the parameters of interest for each iteration and the proportion of the 2000 iterations for which the p-value of the test statistic is less than .05 represents the power for the test of that parameter[41]. The 2000 iterations resulted in 95% confidence interval half-widths of ≤ 2 percentage points for all power calculations reported. Further details on this method can be found elsewhere [21,41-44].

## Results

### Heterogeneity of treatment benefit

Panel A of the Figure shows the functional relationship between net RRR (treatment benefit *minus *treatment harm) and pre-treatment risk (which in a clinical trial is equivalent to the true CER control event rate [CER]) in a hypothetical clinical trial when a treatment decreases 5-year pre-treatment risk by 50% but at a cost of 3 serious treatment-related complications per year per 1000 people treated. As can be seen, even with this relatively large relative treatment-effect and the relatively modest treatment-related harm, net harm will result when the 5-year pre-treatment risk of a study subject is lower than 3%, as the absolute treatment benefit is offset by the absolute treatment-related harm. Thus, net benefit of treatment arises only at pre-treatment risks above 3%. However, there are diminishing improvements in net RRR as pre-treatment risk increases. Note how there is a dramatic improvement in net RRR as pre-treatment risk increases from 2% to 5%, but that further improvements in net RRR above 5% pre-treatment risk are more modest. This is important since subgroup analyses test for differences in net RRR, not differences in ARR.

Panel B shows the statistical power to detect this heterogeneity in net RRR when using conventional subgroup analyses vs. a multivariable risk-stratified approach. As can be seen, if there are 4 independent RF's (OR = 2 for each), adding these 4 RF's into a simple risk index (0-4 RF's) substantially improves statistical power over conventional ("one-variable-at-a-time) subgroup analysis. The improved statistical power of the multivariable approach is merely a by-product of improved risk-stratification. Since a multi-variable model stratifies patients into subgroups with a much wider range of pre-treatment risks, there is more variation in net RRR across treatment groups, and hence, it is easier to detect the underlying heterogeneity in net treatment benefit. In particular, since a multivariable prediction tool is often required to identify low-risk study subjects, [22,28-40] net treatment harm in lower-risk patients can be completely missed if we rely solely on conventional subgroup analysis [22,26].

### The impact of the pre-treatment risk of the study population

Panel B of Figure 1 also shows how the overall pre-treatment risk of the study population increases, the likelihood of detecting treatment-effect heterogeneity diminishes for both the conventional and multivariable approach. However, this is not really due to a change in statistical power, *per se*, but rather a change in the magnitude of true heterogeneity in net RRR within the study population. That is, even if there is substantial variability in pre-treatment risk, if few subjects are close to the point where net RRR is zero (in this case, a pre-treatment risk of 3%), there can be relatively low heterogeneity in net RRR across the study population (once again note the small difference in net RRR between subjects with a 6% vs. a 9% pre-treatment risk).

Since the pre-treatment risk (CER) for the lowest risk subgroup is such an influential factor, for the remainder of our analyses we held the 5-year CER for those with no risk factors constant at 0.75%, so as to examine the influence of other factors.

### The impact of the prevalence and predictiveness of a risk factor on conventional subgroup analysis

The results presented in Table 2 quantify two well known statistical phenomena. First, that there is only a small marginal gain in statistical power when comparison groups have equal sample size (prevalence of a risk factor = 50%) vs. when one group has 3 times the sample size of the other group (prevalence of a risk factor = 25%). For example, for a risk factor with an odds ratio of 2, the statistical power to detect heterogeneity in net RRR had statistical power that was only slightly better when the prevalence of the RF was 50% vs. 25% (power was .22 vs. .21, respectively). However, as the prevalence of a RF falls below 25%, statistical power begins to decline rapidly. Second, Table 2 demonstrates how rare it will be for a conventional subgroup analysis to have good statistical power to detect treatment benefit heterogeneity due to treatment complication rates. For example, even under a near-ideal circumstance (a well powered study in which net RRR varies dramatically between lower vs. higher risk subjects), a subgroup analysis for a RF with an odds of 3.0 and a prevalence of 50% still only has a 44% statistical power. A review of risk prediction tools for commonly occurring outcomes shows that it is rare for a single risk factor to have an independent impact on risk that is greater than 2-fold [22,28-40]. This lack of statistical power is a major reason that most trials attempt to only qualitatively compare the relative risks in the overall trial populations and various subgroups.

### Caveat: conventional subgroup analyses may be useful for detecting heterogeneity in RRR due to differential treatment response

The above conclusion does *not *suggest that looking at individual patient factors in isolation will always have poor statistical power. Although it appears that conventional subgroup analysis will rarely be robust in evaluating the phenomenon being examined in this paper (i.e., heterogeneity in net RRR due to treatment-related harm), examining individual patient factors can be quite important if the variable is a measure of or marker for something that directly modifies the likelihood of treatment response (i.e., directly influences the treatment's RRR). Examples of circumstances in which individual patient attributes would be anticipated to possibly modify treatment effects would include hormone receptor status in a tamoxifen trial, or baseline renin-angiotension measures in a study of ACE inhibitors, instances in which the subgroup variable is related to the treatment's mechanism of action and therefore directly modifies responsiveness to the treatment.

### The impact of adding RF's into a risk index

Table 3 further explores how adding RF's into a risk index (a simple version of a multivariable risk-stratified approach) affects statistical power. We find that combining 3 RF's with effect sizes of RR = 1.5 has poor statistical power for detecting the heterogeneity in net RRR shown in Figure 1; however, this risk index also has very poor discriminant (predictive) ability (area under the ROC [AUROC] = 0.55). As the predictiveness of the risk index is improved (by adding together more risk factors or having RF's of greater effect size), the statistical power for detecting heterogeneity in net RRR increases. In all instances combining risk factors into a risk index substantially improves statistical power compared to looking at RF's individually; however, in this example using a risk index with an AUROC <.6 does not achieve even moderate statistical power. Therefore, it is encouraging that for most common major clinical outcomes, we have validated risk prediction tools with an AUROC ≥ 0.65 [22,28-40]. Since obtaining moderate levels of risk prediction (AUROC > 0.6) usually requires combining at least 4–6 RF's into a prediction tool, [22,28-40]. Table 3 also provides additional evidence suggesting that it will be quite rare for conventional subgroup analysis to have even moderate statistical power for detecting this type of treatment benefit heterogeneity.

### The importance of the accuracy of the weights used for the prediction tool

There is reason to be concerned that the weights used in published prediction tools might be poorly generalizable to different clinical settings or to different patient populations. Therefore, we examined the effect of varying the accuracy of the weights used to construct the prediction tool. As expected the accuracy of the weights used in the risk index substantively affect statistical power; however, the impact was less than we had anticipated (see Table 4). For example, going from perfect weighting of the risk tool to reverse weighting (i.e., those risk factors with a true RR of 1.5 are given a weight of 2.5 and those with a true RR of 2.5 are given a weight of 1.5), decreases statistical power from 82% to 69%. Although this is a meaningful decrease in statistical power, conventional subgroup analysis for an individual RF's with a RR of 2.5 only has a statistical power of 37%. Reverse weighting with the omission of two of the RF's further decreases statistical power, but the decrement is similar to what would be expected given the association between risk model predictiveness (AUROC) and statistical power seen in Table 3. Therefore, under the scenarios studied, the absolute predictiveness of the model and the degree of risk stratification (Figure) appear to be much more important than whether the poor predictiveness is due to: 1) not many RF's known, 2) known RF's missing from the model, or 3) suboptimal weighting of the relative importance of different RF's.

### The impact of the degree of treatment-related complications

Table 5 examines how the probability of treatment-related complications will influence the amount of heterogeneity in net RRR, and thus, the statistical power of subgroup analyses. As expected, the heterogeneity in net RRR increases as the rate of treatment-related complications increases, thus making it easier to detect the underlying phenomenon. However, even a very low rate of treatment-related complications (1–2 adverse events per year per 1000 people treated) can result in important and detectable differential benefit. Of course, the heterogeneity in net RRR will decrease as the pre-treatment risk of the lowest risk study subject's increases, as was demonstrated in the Figure previously.

## Discussion

Although several investigators have laid out a general theoretical justification for multivariable risk-stratified subgroup analysis of clinical trials, [9,11-14,22,23,45] this is the first paper to systematically quantify the degree to which it improves statistical power for detecting heterogeneity in net RRR. In particular, we examined the common circumstance in which multiple risk factors for the main study outcome are known and some portion of treatment-related harm is independent of predicted risk in the absence of treatment (resulting in a more uniform distribution of treatment related complications). We found that when such a phenomenon occurs, that conventional subgroup analyses will rarely have even moderate statistical power to detect heterogeneity in net RRR and that a multivariable approach will generally substantially improve statistical power. Although having at least a moderately predictive tool is important (AUROC > .6), another highly influential factor is whether there are a sufficient number of study subjects who have relatively low risk in the absence of treatment. When the phenomenon studied in our analyses occurs (substantial reduction of pre-treatment-related risk but at a cost of a set amount of serious treatment-related complications), there will usually be greater heterogeneity of net RRR within the moderate-to-low risk subjects, therefore, our ability to detect this phenomenon is heavily influenced by whether we have an adequate number of study subjects spanning this range of risk. This finding has two important implications. First, it reinforces the caution to clinicians about not extrapolating net RRR of a treatment to patients whose risk for the primary outcome is substantially lower than that of the people evaluated in the clinical trial. For example, statin therapy is known to have an excellent risk-benefit ratio (i.e., net RRR) in high CV risk subjects; however, caution should be used in extrapolating those results to low CV risk patients[23]. Second, since low risk subjects can often only be identified by multivariable risk models, these results suggest that multivariable risk-stratified analyses should be conducted routinely whenever an adequate externally-developed prediction tool is available. Otherwise, safety problems occurring in low-risk subjects could be missed. Given potential undetected safety problems and the strong rationale for risk-stratified analysis, we also feel that *post hoc *risk-stratified analyses should be conducted on previously completed clinical trails when such analyses are feasible.

Although there are published risk-prediction tools for most major clinical outcomes, there are some reasons to be concerned about the adequacy of these prediction tools, especially for use in clinical practice. These tools have reported AUROC's that are usually greater than .65; [22,28-40] however, it is unclear how often these tools will maintain their predictiveness when used in other clinical settings and patient populations. Greater attention to developing, adapting and validating risk prediction tools will undoubtedly be needed if risk-stratified analysis is to realize its full impact on improving our understanding of variations in treatment benefits. The challenge of adapting the results of risk-stratified analysis to clinical practice will be even more daunting[9]. A thorough discussion of this topic is beyond the scope of this paper, but it should be understood that conducting and reporting risk-stratified analysis is only a first step in the more complex issue of how best to implement more nuanced and appropriate use of increasingly expensive clinical interventions in day-to-day clinical practice [2,9,11-14].

As mentioned earlier, readers should not assume that our results suggest that conventional subgroup analysis should be abandoned. Conventional subgroup analysis may be poorly suited to detect the phenomenon examined in this study; however, we recommend that conventional subgroup analyses focus on situations in which there is reason to believe that an individual patient attribute may influence the treatments mechanism of action (such as hormone receptor status in breast cancer patients or time since onset of chest pain for heart attack patients). With the advent of pharmocogenetics, highly influential individual factors might increasingly be found that modify treatment effects. However, for examining the impact of treatment-related risk, risk factors should be combined together using a previously developed and validated risk prediction tool whenever possible, with special attention to the net RRR in lower risk subjects. Even when net RRR is homogeneous across the study population, such analyses will highlight how absolute risk reduction and number needed to treat vary across the study population.

## Conclusion

Although all subgroup comparisons require sound justification and cautious interpretation, the improvements in statistical power of a risk-stratified approach for detecting significant heterogeneity in treatment efficacy and safety appear to be quite substantial under many likely clinical scenarios. We conclude that if an adequate externally-developed risk prediction tool is available that a multivariable risk-stratified analysis should be conducted and reported along with the main results of the clinical trial.

## Competing interests

The author(s) declare that they have no competing interests.

## Authors' contributions

TH created the initial statistical code for the simulation structure and consulted on all aspects of the analysis. SV reviewed the simulation results and contributed to the analysis plan. DK was instrumental to the conception of the study and made major contributions to the analysis plan. RH conceived of the study, developed the analysis plan, conducted the statistical analysis and drafted the manuscript. All authors participated in the design of the study, and read and approved the final manuscript.

**Table 1:** Results of conventional vs. risk-stratified analyses when treatment decreases pre-treatment risk by 50% but at a cost of 3 serious adverse events per year of treatment (6 independent risk factors (RF's) exist, each with a prevalence of 25%)

	True Control Event Rate (CER)	True Relative Risk Reduction (RRR)	True Number Needed to Treat (NNT)	Statistical Power of Subgroup Comparison*
N = 8,800 (% of study population)	For 5-Year Follow-up			P < 0.05

**Conventional Subgroup Comparison**				
Risk factor absent (75%)	2.2	-.19†	-239†	.23
Risk factor present (25%]	4.2	.13	183	

**Risk Index **(Dichotomized measure)				
0–1 Risk factors (53.4%)	1.4	-.57†	-125†	.72
≥ 2 Risk factors (46.6%)	4.4	.16	143	

**Risk Index **(continuous measure)‡				
0 Risk factors (17.8%)	0.75	-1.59†	-88†	.83
1 Risk factors (35.6%)	1.5	-.51†	-132†	
2 Risk factors (29.7%)	3.0	-.02	-1936	
3 Risk factors (13.2%)	6.0	.21	83	
≥ 4 Risk factors (3.7%)	12.8	.35	24	

* For the subgroup comparisons, the statistical comparison tests whether the subgroup with the risk factor receives more or less benefit (two-tailed testing) than the subgroup without the risk factor (testing for an interaction between the risk factor and intervention [treatment vs. control] in a logistic regression model. ^21 ^For example, the conventional subgroup comparison had a statistical power of 23% for detecting that those with the risk factor had a greater relative benefit from treatment than those without the risk factor.

† The minus sign denotes that treatment had net harm, rather than benefit.

‡ Area Under the Receiver Operator Characteristic (AUROC) curve for the Risk Index was 0.65.

**Table 2:** Statistical power when subgroup analysis is done for each risk factor one-at-a-time

		Risk Factor Prevalence *		
	
		**10%**	**25%**	**50%**
	
		*Statistical Power of Subgroup Comparison *†		
**Risk Factor Effect Size (Odds Ratio)**	**1.5**	**.08**	**.12**	**.13**
	**2.0**	**.13**	**.21**	**.22**
	**3.0**	**.26**	**.41**	**.44**

* Risk Factor is one of six independent risk factors, with the other five risk factors having a prevalence of 25% and an odds ratio of 2 when the 5-year CER for those without any risk factors is .75% (see Table 1 and Figure).

† Statistical subgroup comparisons tests the power to detect whether those with the risk factor had a greater relative benefit from treatment than those without the risk factor.

**Table 3:** Table 3. Statistical power when risk factors are combined into a risk score

		**Number of Risk Factors in the Risk Score**		
	
		**1**	**3**	**6**
	
		*Statistical Power for Risk Index (AUROC) *†		
**Risk Factor Effect Size (Odds Ratio)**	**1.5**	**.12 (.53)**	**.27 (.55)**	**.45 (.58)**
	**2.0**	**.21 (.54)**	**.61 (.59)**	**.83 (.65)**
	**3.0**	**.41 (.57)**	**.92 (.67)**	**.93 (.76)**

* In each instance all risk factor have a prevalence of 25% and the 5-year CER for those without any risk factors is .75% (see Table 1 and Figure).

† Statistical subgroup comparisons tests the power to detect whether the treatment's relative benefit varies as a function of the risk score. The Area Under the Receiver Operator Characteristic (AUROC) curve is a measure of the overall predictiveness of a model for predicting a dichotomous outcome (i.e, event occurred vs. event did not occur).

**Table 4:** The importance of the accuracy of the weighting of the risk prediction tool

	RF's #1 & #2*	RF's #3 & #4*	RF's #5 & #6*	**Risk Index's Predictiveness (AUROC) **†	**Statistical Power (p < 0.05)‡**
**True Predictiveness**	**1.5**	**2.0**	**2.5**	**-**	**-**
**Perfect Weighting**	**1.5**	**2.0**	**2.5**	**.69**	**.82**
**Uniform Weighting**	**1**	**1**	**1**	**.66**	**.75**
**Reverse Weighting**	**2.5**	**2.0**	**1.5**	**.63**	**.69**
**Incomplete Reverse Weighting**	**2.5**	**0.0**	**1.5**	**.60**	**.51**

* Each of the 6 risk factors (RF's) has a prevalence of 25% and the 5-year CER for those without any risk factors is .75% (see Table 1 and Figure). The true predictiveness (relative risk) of the risk factor is shown as well as the relative weight used in the risk index.

† The Area Under the Receiver Operator Characteristic (AUROC) curve is a measure of the overall predictiveness of a model for predicting a dichotomous outcome (i.e, event occurred vs. event did not occur).

‡ Statistical subgroup comparisons tests the power to detect whether the treatment's relative benefit varies as a function of the risk score.

**Table 5:** How does the degree of treatment-related risk influence the statistical power of the subgroup analysis? *

**Annual Risk of Treatment-Related Adverse Events**	Conventional "One-RF-at-a-Time" Subgroup Comparison*	Multivariable Risk Stratified Analysis*
*(Events per 1000 patient-years)*	*Statistical Power *†	

1	6%	39%
2	11%	59%
3	21%	83%
4	28%	92%

* There are 6 risk factors (RF's) that each have a prevalence of 25% and a relative risk of 2, and the 5-year CER for those without any risk factors is .75% (see Table 1 and Figure).

† Statistical subgroup comparisons tests the power to detect whether the treatment's relative benefit varies as a function of the presence of the RF (for conventional subgroup analysis) or the risk score (for the multivariable risk-stratified analysis)

**Table 6:** Glossary

**Risk-Benefit Prediction Model **– Same as a Risk Prediction Model except factors that predict benefit of a specific treatment and those that predict harm from the treatment are also included.
**Control Event Rate (CER) **– Rate of bad outcomes in the control group.^46^
**Pre-treatment risk **– Risk of bad outcomes in the absence of treatment. In a clinical trial, pre-treatment risk is equivalent to the true control event rate (CER).
**Experimental Event Rate (EER) **– Rate of bad outcomes in the intervention group. ^46^
**Relative Risk Reduction (RRR) **– The proportional reduction in the rate of bad events between experiment (experimental event rate [EER]) and control (control event rate [CER]) patients in a trial, calculated as |EER – CER|/CER. ^46^
**Absolute Risk Reduction (ARR) **– The absolute arithmetic difference in event rates, |EER – CER|. ^46^
**Number Needed to Treat (NNT) **– The number of patients who need to be treated to prevent 1 additional bad outcome; calculated as 1/ARR.

Risk Prediction Model – Predicting overall risk based upon combining information from multiple risk factors. Prediction models can be presented as full regression prediction model (such as predicted probability of death using APACHE), as a simple risk index (such as predicting birth outcomes using a 10 point APGAR score) or condensed into risk categories (low, medium, high peri-operative risk).

## Pre-publication history

The pre-publication history for this paper can be accessed here:


